# The Downsides of Cognitive Enhancement

**DOI:** 10.1177/1073858420945971

**Published:** 2020-07-30

**Authors:** Lorenza S. Colzato, Bernhard Hommel, Christian Beste

**Affiliations:** 1 Cognitive Neurophysiology, Department of Child and Adolescent Psychiatry, Faculty of Medicine, TU Dresden, Dresden, Germany; 2 Department of Cognitive Psychology, Institute of Cognitive Neuroscience, Faculty of Psychology, Ruhr University Bochum, Bochum, Germany; 3 Cognitive Psychology, Faculty of Psychology, Shandong Normal University, Jinan, China; 4 Cognitive Psychology Unit & Leiden Institute for Brain and Cognition, Leiden University, Leiden, Netherlands

**Keywords:** cognitive enhancement, neural competition, neurotransmitters, tDCS, drugs

## Abstract

Cognitive enhancement is becoming progressively popular as a subject of scientific investigation and by the public, although possible adverse effects are not sufficiently understood. We call for cognitive enhancement to build on more specific, mechanistic theories given that a-theoretical approaches to cognitive enhancement are both a cause and a consequence of a strong, if not exclusive focus on the benefits of procedures suited to enhance human cognition. We focus on downsides of cognitive enhancement and suggest that every attempt to enhance human cognition needs to deal with two basic principles: the neuro-competition principle and the nonlinearity principle. We discuss the possibility of both principles in light of recent attempts to improve human cognition by means of transcranial direct current stimulation, a well-established brain stimulation method, and clinically relevant nootropic drugs. We propose that much stronger emphasis on mechanistic theorizing is necessary in guiding future research on both the upsides and the downsides of cognitive enhancement.

## Introduction

“It’s not about being the best. It’s about being better than you were yesterday”—this motivational quote may be found on the wall of many gyms and offices, and it seems to capture the essence of cognitive enhancement: to reach one’s personal (physical or mental) best without necessarily outperforming others. Hence, cognitive enhancement can be defined as the employment of any (legal) ways (e.g., through cognitive training, brain stimulation, nootropics, video games, or neurofeedback) to enhance human cognition and action in healthy individuals (Bostrom and Sandberg 2009). Meta-analyses show that different enhancing techniques seem to have a selective impact on enhancing cognition in healthy humans across the lifespan. Whereas physical exercise seems to be particularly effective in children (Verburgh and others 2014), noninvasive brain stimulation is apparently a good candidate to compensate for cognitive decline in aging (Hsu and others 2015), and playing action video games an efficient way to improve cognitive performance in healthy young adults (Wang and others 2016). In the field of cognitive enhancement, the focus on healthy people rely on the goal to boost, among others, working memory, which is essential for achievements in school and working environments (Diamond 2013) but also for successful aging (Rowe and Kahn 1997). Indeed, boosting the accuracy of working memory can help, for example, the elderly to better remember the items to buy at the grocery store (Cowan 2010).

The appeal of cognitive enhancement has a strong economic aspect: the progressively ageing population in Westerns societies increasingly challenges the sustainability of the welfare system, which raises the question how aging can be made less costly (James 1995). Cognitive enhancement can be useful in this respect by delaying or compensating for the cognitive decline of senior citizens (Kramer and Willis 2002), which prolongs the time older individuals can autonomously reside in their homes—which combines increasing individual well-being with reducing societal welfare costs (Diener and Seligman 2004). Conversely, it is sometimes thought that enhancing children and adolescents can help accelerate the learning curve toward educational and professional success (Salkever 1995), which in turn makes education cheaper and education-dependent problematic behavior less likely (Kremer and others 2016).

In addition to financial reasons, ideological considerations promote the relevance of cognitive enhancement. Especially Western societies are facing an apparently unstoppable development toward individualism, which highlights and is based on individual differences, at the expense of shared and reciprocal societal values (Santos and others 2017). These trends are closely related to a revival of a sociopolitical *consensus gentium* toward right wing neoliberalism in many European countries and the United States, which promotes a monetary perspective on societal institutions (presumably resulting in the dismantling of the welfare system) and a utilitarian view of the human being as creator of his or her own destiny (Fenger 2018). While by far not all reasons to engage in and promote cognitive enhancement rely on such neoliberal considerations, the field of cognitive enhancement has profited from these steady developments toward cost-effective policies of the welfare system and more individual(istic) freedom and responsibility (Kelly and Morar 2019). For these and other reasons, we think that we are only seeing the beginning of attempts to enhance human cognition.

The availability of methods to enhance human cognition raises many questions, and some have led to heated debates about the ethical and societal implications of cognitive enhancement (Bostrom and Sandberg 2009). As important as these discussions are for both ethical and societal reasons (Farah 2005; Kadosh and others 2012; Mohamed 2014; Sahakian and Morein-Zamir 2011; Turner and Sahakian 2006), they tend to overshadow other, more scientifically relevant implications of cognitive enhancement that we would like to focus on here. Specifically, we argue that the enthusiasm about the possible pros of attempts to improve the cognitive abilities and skills of individuals has led to a widespread neglect of the possible cons. As we will try to show, truly understanding the possibly delicate relationship between pros and cons of enhancement and enhancement techniques requires much deeper insights into the functional and neural mechanisms underlying human cognition than many of the current approaches are based on.

Indeed, most current approaches are rather explorative and rarely guided by systematic theoretical frameworks and mechanistic insights into the functioning of the human cognitive system (Colzato and Hommel 2016). We believe that this mainly a-theoretical approach to cognitive enhancement is both a cause and a consequence of a strong, if not exclusive focus on the benefits of procedures suited to enhance human cognition. In contrast to this emphasis of the benefits of enhancement techniques, of which many indeed exist, we would like to call for a more balanced view that also takes the negative side of cognitive enhancement into account. Like there is no light without shadow, cognitive enhancement may not only be linked to mental gains but also to potential mental costs. These mental costs are, however, often neglected in contemporary use and research practices in the field of cognitive enhancement. In the following, we argue that mental costs are likely if two basic principles of cognitive enhancement are not sufficiently heeded: the principle of neural competition and the principle of nonlinearity.

## Gains Come with Losses: The Principle of Neural Competition

**Figure 1.**
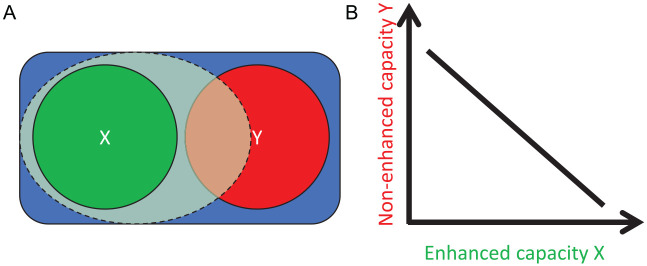
Illustration of the neural-competition principle. (A) The available neural/cognitive resources are shown in blue, the resources normally used by capacity X are shown in green, and the resources normally used by capacity Y are shown in red. Enhancing X is likely to increase the capacity used by X (see light-green area), which leaves less capacity to Y. (B) The result of this relationship: the more capacity X is enhanced, the more the nonenhanced capacity Y is impaired.

One of the key characteristics of the human (but not only the human) brain is its competitive nature (Cisek 2019): Neurons and neural networks compete for the representation and processing of environmental and internal information (Güntürkün and others 2000), attention to endogenous or exogenous events and representations (Fornito and others 2012), or the selection of actions (Bogacz 2007; Cisek 2007), which directly implies that where there are winners of such kinds of competition there must be losers. On a related note, Kurzban and others (2013) propose that experiencing fatigue is linked to cost/benefit signals coded in the prefrontal cortex, the capacity of which is limited by the number of computational operations that it is able to carry out at any given time. Given that the brain and its subsystems are both logically and empirically limited in capacity, improving, or increasing the efficiency of one particular function or process or representation must imply some kind of loss associated with another function or process or representation. However, at present, the potentially inevitable loss is not considered in the scientific discussion of cognitive enhancement. The consideration that gains should come with losses is also consistent with assumptions based on game theory. As pointed out by Brem and others (2014), game theory suggests that one party’s gain corresponds to another party’s loss, in such a way that the net change in terms of benefit is always zero. Applying this *net zero-sum framework* to the human brain suggests that enhancing one cognitive function (i.e., generating neural gains) would be likely to result in neural losses, so that the improvement of the targeted cognitive function would be expected to impair other cognitive functions (see Fig. 1). In other words, cognitive enhancement may be impossible without cognitive cost. As postulated by Brem and others (2014), it is important to keep in mind that cognitive effects are not exclusively due to direct effects of a single stimulated brain area or neurotransmitter but the result of the activation or inhibition of an entire interactive brain network. Furthermore, even if gains in a cognitive domain is found, a loss in another domain might not always be detectable because the gain does not reach a certain threshold (Aston-Jones and Cohen 2005; Servan-Schreiber and others 1990) or simply because other cognitive functions were not assessed.

A concrete application of the net zero-sum concept is the metacontrol hypothesis of cognitive control (Beste and others 2018; Goschke 2000; Hommel and Colzato 2017). The hypothesis is based on the idea that cognitive control is not a unitary function but an emerging property of the interaction of (presumably prefrontal) (Stokes 2015) systems promoting cognitive persistence, including focusing on one goal and on relevant information, and (presumably striatal) (Kehagia and others 2010) systems promoting cognitive flexibility, as needed, for instance, for switching to other plans, opening up for other opportunities, and considering a broader range of possibilities. The actual challenge of cognitive control would thus consist in determining the appropriate balance between cognitive persistence and cognitive flexibility (Goschke and Bolte 2014; Hommel, 2015). Along the same lines, it has been argued that adaptive behavior requires finding the right balance between exploitation and exploration (Hills and others 2015) and between speed and accuracy (Bogacz and others 2010). The need to balance between these kinds of processing modes results from the fact that they exclude each other: the better one manages to focus on task-relevant information, the more likely one is to ignore information that is task-irrelevant but might signal a more interesting, rewarding, or appropriate action opportunity—and vice versa. Importantly, this means that any attempt to improve people’s ability to engage in one of the alternative processing modes could be expected to impair their ability to engage in the other. The ability to distinguish what is signal and what is noise is crucial for an effective balance between cognitive persistence and cognitive flexibility. The processing of neural noise and adaptation of the signal-to-noise ratio (SNR) during information processing (Adelhöfer and others 2018; Pertermann and others 2019b; Pertermann and others 2019a; Salinas and Thier 2000; Servan-Schreiber and others 1990) and motor levels (Greenhouse and others 2015; Thura and Cisek 2016) is the most plausible neural candidate underlying the metacontrol hypothesis of cognitive control. Enhancing cognitive persistence requires the distinction between relevant and irrelevant information which underlies less noise (i.e., high SNR) and better/more stable cognitive performance across different cognitive domains (Aston-Jones and Cohen 2005; Bensmann and others 2018; Bensmann and others 2019). In contrast, higher noise (i.e., low SNR) might produce more behavioral variability (Gureckis and Love 2009) supporting cognitive flexibility. Hence, the trade-off between enhanced and nonenhanced cognitive functions might depend on the SNR: high SNR might enhance cognitive persistence but at the costs of cognitive flexibility and the low SNR the other way round.

Some evidence for a tradeoff between enhanced and non-enhanced cognitive functions has been obtained by means of transcranial direct current stimulation (tDCS)—a recognized cognitive enhancer (Filmer and others 2014) that is freely available to the general public, be it through commercial devices recently put on the market or do-it-yourself devices easily assembled at home. tDCS increases brain excitability through weak, direct electric currents via electrodes positioned on the skull, ideally over brain areas related to the to-be-enhanced function (Nitsche and Paulus 2000). If the net zero-sum logic applies, stimulating the brain to achieve neural and functional gains should produce neural and functional losses. Indeed, Iuculano and Cohen Kadosh (2013) demonstrated that cognitive enhancement and impairment can be achieved by means of the same stimulation protocol. In particular, they showed that stimulating one brain area can facilitate numerical learning but impair automaticity of the learned material, while stimulating another brain area enhances automaticity while impairing the learning process. Hence, cognitive enhancement of one function can take place at the expense of another function. Consistent with this idea, other noninvasive brain stimulation protocols (Najib & Pascual-Leone 2011) found similar results regarding other cognitive functions such as set-shifting performance (Leite and others 2011), mental calculation (Krause and others 2019), visual spatial attention (Hilgetag and others 2001; Jin and Hilgetag 2008), declarative and procedural consolidation (Galea and others 2009), and verbal encoding (Kahn and others 2005). From a neurobiological perspective, it has been shown that tDCS can mimic the effects of neurotransmitters (particularly norepinephrine), which is why neuromodulatory effects exerted by such transmitters are unlikely to take place once a tDCS intervention has been conducted (Adelhöfer and others 2019).

Other observations of the same sort were obtained by using clinically relevant nootropic drugs to boost cognition, like the widely used drugs modafinil, methylphenidate, and amphetamine, but other psychoactive substances such as benzodiazepines and glucocorticoids are also relevant to consider. Modafinil has already been established as cognitive enhancer in various occupations necessitating protracted wakefulness, such as soldiers and medical and paramedical personnel. Consistent with this picture, about 90% of modafinil consumers are healthy people with no sleep disorders who are using the medication to increase their attentional focus under fatigue (Baranski and others 2004). In sleep-deprived individuals, research has demonstrated that multiple small doses of modafinil over time or a single, large dose can successfully maintain cognitive performance and restore cognitive functioning to near-baseline levels (Stivalet and others 1998). However, the enhancing effects come at the cost of subjective overconfidence: people evaluate their own cognitive performance to be better than their actual performance. That is, using modafinil impairs effective self-assessment and self-monitoring, which among other things might make unrealistic risk-taking more likely (Baranski and Pigeau 1997; Batéjat and Lagarde 1999; Gurtman and others 2008). Along the same lines, methylphenidate (primarily known by its brand name Ritalin) is not only used as pharmacological treatment for attention deficit/hyperactivity disorder (ADHD), but its nonmedical use has grown among healthy people, especially college students to enhance academic achievement. The drug is known to increase the stability of mental representations, but, at the same time to worsen the capability to flexibly update such representations (Fallon and others 2017). Notably, these effects strongly depend on prior learning experience (Mückschel and others 2020a; Mückschel and others 2020b) and thus a critical factor modulating the SNR and hence a balance between cognitive persistence and cognitive flexibility. In fact, prior learning can invert the effects of methylphenidate (Mückschel and others 2020a; Mückschel and others 2020b). This suggests that the drug operates by biasing the person’s metacontrol state toward persistence (cf., Hommel, 2015), thereby preventing cognitive flexibility.

## People Are Not the Same: The Principle of Nonlinearity

**Figure 2.**
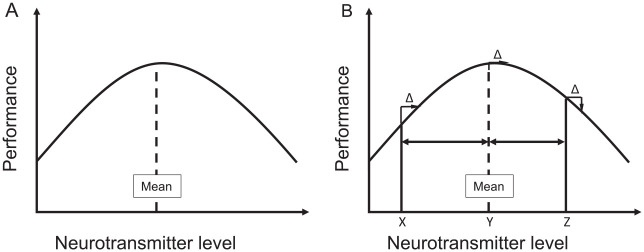
Illustration of the nonlinearity principle. (A) Performance tends to relate to neurotransmitter levels in a nonlinear inverted-U-shaped fashion, so that medium levels are associated with best performance. (B) Enhancing neurotransmitter levels can boost or impair performance in different individuals, depending on their original level. Here, person X has a low level, Y a medium level, and Z a high level of the respective neurotransmitter. Increasing the level by the amount of Δ will thus improve performance in X, have little effect on Y, and impair performance in Z.

Enhancement techniques often operate, either directly or indirectly, by affecting neurotransmitters, like dopamine, noradrenaline, or serotonin (Husain and Mehta 2011). On the one hand, this explains why enhancement can show some degree of far transfer, in the sense that the intervention can affect various kinds of performance. On the other hand, however, neurotransmitter levels are very unlikely to relate to cognitive performance in a linear fashion. This is also suggested by the interactive effects between drugs (e.g., methylphenidate) and prior learning experience (Mückschel and others 2020a; Mückschel and others 2020b). Indeed, it is commonly medium neurotransmitter levels that are related to the best performance, as implied by the findings shown in Figure 2A, while very low and very high levels are often associated with poor performance or even pathologies (Cools and D’Esposito 2011). This has two important implications for cognitive enhancement. For one, it means that whether a given intervention improves or impairs the performance of a given person depends on the base level of this person: the closer an individual is to its theoretical optimum, the more likely an intervention will impair performance. For another, it means that whether a given intervention produces gains or losses depends on its dosage, as well as on the individual “starting point” that can depend on prior learning experience. Figure 2B combines these two implications, which have been tested by means of a divergent-thinking (brainstorming) task that has been argued to rely on striatal dopamine (Ashby and others 1999; Hommel and Colzato 2017). For one, performance in this task was found to relate to individual differences in spontaneous eye blink rates (a clinical marker of striatal dopamine levels; Karson 1983) by means of an inverted-U-shape function: individuals with medium blink rates clearly outcompeted individuals with low or high blink rates (Akbari Chermahini and Hommel 2010). For another, inducing positive mood (a manipulation that is assumed to increase striatal dopamine levels; Akbari Chermahini and Hommel 2012a) improved performance in low blinkers while not affecting performance in medium blinkers (Akbari Chermahini and Hommel 2012b).

Along similar lines, Sarkar and others (2014) employed tDCS to study how the trade-off between enhancing some cognitive functions and impairing others depends on individual differences. Taking into account individual differences is crucial because, as proposed by Krause and colleagues (Krause and others 2013; Krause and Cohen Kadosh 2014), the critical equilibrium between neural excitation and inhibition, and corresponding cognitive enhancement or impairment, varies between specific brain regions and individual factors, such as biological sex and hormonal concentrations, current brain state, preexisting cortical excitability, and age. That is, following the logic of an inverted U-shaped dose-effect relationship between neural excitation/inhibition and cognitive performance, the administration of anodal stimulation might produce a neural hyperexcitation and related cognitive costs in people with elevated regional excitability, but an optimal neural excitation and associated cognitive enhancement in people with low regional excitability. Furthermore, it is important to keep in mind that different brain regions can display different kinds of equilibrium between neural excitation and inhibition, so that identical stimulation parameters in another brain area might produce opposite cognitive outcomes. In sum, following a net zero-sum logic, there is increasing evidence (e.g., Iuculano and Kadosh, 2013; Sarkar and others 2014) that cognitive enhancement and cognitive impairment can take place in parallel and that the equilibrium between the two relies on individual biological traits.

Individual differences also play a key role in the enhancing effects of Adderall, an amphetaminergic drug used as cognitive enhancer: amphetamine intake enhances performance in people with low, but impairs it in people with high baseline functioning (Farah and others 2009). Similar findings were obtained from studies of dopamine-relevant genetic profiles, as dopamine is the main neurotransmitter enhanced by this drug: individuals with a genetic profile that is assumed to be associated with less efficient dopaminergic processing have been found to benefit from the intake of amphetamine, while no such effects were found for individuals with profiles associated with more efficient dopaminergic processing (Ilieva and others 2013; Mattay and others 2003).

Taken altogether, the available findings are in line with the net zero-sum concept by converging in suggesting that cognitive enhancement and cognitive costs can coexist and that the balance between the two relies on several factors such as individual differences and learning, but potentially many more (for some evolutionary considerations, see Hills and Hertwig 2011). In addition, the balance between cognitive enhancement and impairment also seems to depend on whether the intervention is carried out before or after a certain cognitive function takes place. For instance, valium, the most widely used benzodiazepine, impairs memory when provided before memory encoding (so-called anterograde amnesia) but promotes memory when provided after encoding (so-called retrograde facilitation; Hinrichs and others 1984). Similarly, the administration of adrenal glucocorticoids cortisol can enhance or impair memory, depending on whether it takes place before learning or before retrieval (Het and others 2005). These observations suggest that successful cognitive enhancement requires a finer-grained theoretical understanding of the to-be-enhanced processes than commonly found in enhancement studies. Interventions are likely to target particular functional or neural processes that may subserve a particular function, such as encoding and retrieval both subserve memory, but not the function as an undifferentiated whole. It is thus unlikely that interventions will be found that are good for perception, attention, memory, or thinking in general. More successful will be approaches that go deeper into the functional and neural underpinnings of each individual process that contributes to the overall function.

## Conclusions

Current approaches to cognitive enhancement in humans have focused, too much we argue, on the positive aspects of enhancement techniques. This has been a consequence of pragmatic, effect-oriented research that was lacking theoretical guidance and insight into neural mechanisms. Taking the net zero-sum logic into account, we have provided a few, yet selective proofs-of-principle that cognitive enhancement can come with cognitive impairments. While we do not deny that many positive enhancement effects exist, they are likely to be accompanied by negative aspects. In particular, we argue that any attempt to enhance human cognition needs to consider two basic principles: the neural-competition principle and the nonlinearity principle. Given the competitive nature of the brain, gains are likely to come with losses, and we have discussed findings from tDCS and drug studies supporting this assumption. The balance between gains and losses is also likely to be moderated by individual differences, targeted process, and time of administration—factors that are often neglected in enhancement studies. The mere fact that losses are possible should not be worrying, as the importance of gains and losses depend on the problem and the goals of the respective individual. In particular cases and for instance in clinical practice or cognitive rehabilitation, possible costs may well be outweighed by important benefits (Chang and others 2018; Husain and Mehta 2011). Nevertheless, the free availability of enhancers puts a lot of responsibility on science to provide a more comprehensive picture of the pros and cons of particular enhancement techniques. Taking this responsibility, we argue, requires much stronger emphasis on mechanistic theorizing in guiding future research on both the upsides and the downsides of cognitive enhancement. There are, currently, only a few theoretical approaches that can provide an indication of the levels (or cognitive functions) at which antagonistic effects of cognitive enhancement in the sense of a net zero-sum framework can be expected. Here, the concept of metacontrol could be particularly helpful.
